# Dynamics of two-step reversible enzymatic reaction under fractional derivative with Mittag-Leffler Kernel

**DOI:** 10.1371/journal.pone.0277806

**Published:** 2023-03-23

**Authors:** Maryam Khan, Zubair Ahmad, Farhad Ali, Naveed Khan, Ilyas Khan, Kottakkaran Sooppy Nisar

**Affiliations:** 1 Department of Mathematics, City University of Science and Information Technology, Peshawar, Khyber Pakhtunkhwa, Pakistan; 2 Department of Mathematics, College of Science Al-Zulfi, Majmaah University, Al-Majmaah, Saudi Arabia; 3 Department of Mathematics, College of Arts and Science, Prince Sattam Bin Abdulaziz University, Wadi Al Dawasir, Saudi Arabia; Hodeidah University, YEMEN

## Abstract

Chemical kinetics is a branch of chemistry that is founded on understanding chemical reaction rates. Chemical kinetics relates many aspects of cosmology, geology, and even in some cases of, psychology. There is a need for mathematical modelling of these chemical reactions. Therefore, the present research is based on chemical kinetics-based modelling and dynamics of enzyme processes. This research looks at the two-step substrate-enzyme reversible response. In the two step-reversible reactions, substrate combines with enzymes which is further converted into products with two steps. The model is displayed through the flow chart, which is then transformed into ODEs. The Atangana-Baleanu time-fractional operator and the Mittag-Leffler kernel are used to convert the original set of highly nonlinear coupled integer order ordinary differential equations into a fractional-order model. Additionally, it is shown that the solution to the investigated fractional model is unique, limited, and may be represented by its response velocity. A numerical scheme, also known as the Atangana-Toufik method, based on Newton polynomial interpolation technique via MATLAB software, is adopted to find the graphical results. The dynamics of reaction against different reaction rates are presented through various figures. It is observed that the forward reaction rates increase the reaction speed while backward reaction rates reduce it.

## 1. Introduction

Catalysts (generally proteins) are molecules that convert substrates into products. During the reaction, enzymes do not change. Catalytic power, specificity, and regulation are the most important characteristics. The catalytic action of enzymes involves lowering the reaction free energy of activation by catalyzing the transformation of substrates into products. For example, enzymes can overcome charge repulsions, facilitating the formation of stronger chemical bonds between molecules exposed to each other. It is possible that the enzyme may cause a specific bond to be more difficult to break if the reaction requires breaking an existing bond. Enzymes are extremely good at speeding up biological processes, often by a factor of 10 million or more. They’re also quite specialized, frequently catalyzing only one or a few closely related substrate reactions. As a result of enzyme-catalyzed chemical reactions, enzyme kinetics is studied [1]. The enzyme kinetics is used to determine the reaction rate and investigate the consequences of changing the reaction conditions. The catalytic mechanism of an enzyme and its significance in metabolism can be illuminated by studying its kinetics in this way.

Furthermore, how its behaviour is influenced and how a medicine or modifier (inhibitor or activator) would influence the rate. Miłek [2]. provided several of the more complex reaction schemes, including one that uses an enzyme to catalyze the process. According to Alberty [3] enzymes are the most diverse and specific catalysts. They catalyzed reactions at incredible velocities at neutral pH and low temperatures. Using the Michaelis-Menten equation (a very basic and common substrate-enzyme reaction) and three additional approximations, Cha [4] analyzed the inefficiencies associated with the genuine rate equation, which takes into account the free substrate being drained by the enzyme. Wong [5] investigated reaction steady-state phases based on Michaelis and Menten’s enzymic process. Wald *et al*. [6] investigated the enzymatic degradation of cellulose with the kinetic properties. Urban et al. [7] analyzed the new arising trends in the development of enzymatic microreactors. Najafpour and Shan [8] reported the enzymatic hydrolysis of molasses using glucoamylase. Rigouin *et al*. [9] discussed biochemical methods for the detection of depolymerization.

In the field of mathematical physiology and biochemistry, different researchers reported their contributions by using mathematical tools in the field of chemical kinetics and enzyme mechanisms. For example, Gan *et al*. [10] reported an experimental and analytical investigation of enzymatic cellulose hydrolysis reaction kinetics. Moreover, they analyzed the impact of varying dynamics and the interaction of enzymes with cellulose substrates and products. Savageau [11] described how metabolism is controlled in a biochemical system through the same mathematical description. Atherton *et al*. [11] presented the statistical sensitivity for the chemical kinetics of different reactions. Shen and Larter [12] used typical nonlinear dynamics techniques to investigate two chemical kinetic models in order to establish the situations in which substrate inhibition kinetics can cause oscillations. Masel [13] explained the rate of chemical kinetics and catalysis trajectory calculation to calculate different reaction rates. Wu *et al*. [14] investigated the enzyme-substrate interaction in the presence of functionalized gold nanoparticles by changing the rate constants.

Fractional models gain the attention of many researchers in real-world problems. As it is a powerful mechanism to incorporate the memory effect and crossover behavior in physical problems. Recent studies revealed that fractional derivatives can best explain physical problems as compared to integer-order derivatives. Because of their wide variety of uses in several disciplines of research, different researchers used the idea of fractional calculus in different fields of sciences such as biomathematics, infectious diseases, integrated circuits, fluid flow problems, nanofluids, social problems and dynamical systems [15–27]. A new type of fractional sums and differences called the discrete weighted fractional operators are presented by Abdeljawad *et al*. [28]. Joshi and Jha [29] discuss a mathematical calcium model is developed in the form of the Hilfer fractional reaction-diffusion equation to examine the calcium diffusion in the cells. Abdo [30] discussed some existence results for at least one continuous solution for generalized fractional quadratic functional integral equations. Similarly, the kinetics of drying process of soybean via the fractional operator of Mittage-Leffler operator was studied by Ozarslan and Bas [31]. The wind influenced projectile motion with the fractional operator of the Mittage-Leffler kernel was analyzed by Ozarslan [32]. The mathematical analysis of COVID-19 via fractional differential operator for the case of Pakistan was carried out by Naik [33]. Hammouch et al. [34] analyzed the chaotic system with the variable order fractional differential operator. Keeping in mind these applications of fractional calculus in different research fields, some researchers also conducted their analysis in the field of chemical kinetics [35–38]. Ahmad *et al*. [35] reported chemical kinetics dynamics of cooperative reactions via two different fractional operators of singular and non-singular kernels. Toledo *et al*. [36] explored the fractional model of the principal transport mechanism of biological mechanism. Alshbool and Bernstein [37] investigated the fractional-order basic enzyme reactions via the polynomial technique. Singh *et al*. [38] analyzed the chemical kinetics of the fractional model via the Atangana-Baleanu derivative with fractional order having Mittage–Leffler type kernel.

To the best of the author’s knowledge, no study has been reported to analyze the mathematical model for the dynamics of two-step reversible biochemical reactions. Therefore, the current study contains the dynamics of chemical kinetics of two-step reactions. In order to get the product, a forward-backward two-step enzyme-substrate reaction has been considered which is first portrayed through a flow chart which is further transformed to a system of nonlinear coupled ODEs under the law of mass action. In order to generalize the integer order model, Atangana-Baleanu fractional derivative which has a non-local and non-singular kernel i.e., Mittag-Leffler kernel in order to observe the dynamics in a more complicated fashion and to include the memory effect and crossover behaviour. The fractional model’s boundedness, existence, and uniqueness have been analyzed theoretically. As the exact solutions of such highly nonlinear coupled problems are not possible, a numerical scheme based on the Newton polynomial technique is adopted to find the graphical results. The effect of different reaction rates has been depicted through significant graphs.

## 2. Mathematical preliminaries

In this portion, some important ideas and prepositions will be beneficial to study the fractional-order chemical enzymatic kinetics [28, 39, 40].

**Definition 2.1**: In the case of a continuous function $f(t)\in C^{n}[0,T]$, with a fractional parameter *γ*, The Atangana-Baleanu time-fractional operator in the Caputo sense (ABC) is denoted by the following notation:

$${}_{a}^{ABC}{℘}_{t}^{\gamma }\psi (t)=\frac{N(\gamma )}{1-\gamma }\int_{a}^{t}E_{\gamma }(\frac{-\gamma (t-\omega {)}^{\gamma }}{1-\gamma })\psi^{'}(\omega )d\omega ,\;\mathrm{For}\;0<\gamma <1.$$ (1)

The normalization function is *N*(*γ*), while the Mittage-Leffler function is *E*_*γ*_(.).

**Definition 2.2:** For the ABC fractional derivative, the integral operator is:

$${}_{a}^{ABC}I_{t}^{\gamma }f(t)=\frac{1-\gamma }{N(\gamma )}f(t)+\frac{\gamma }{N(\gamma )\Gamma (\gamma )}\int_{a}^{t}{\left(t-\zeta \right)}^{\gamma -1}f(\zeta )d\zeta .$$ (2)

**Definition 2.3:** Toufik and Atangana define the numerical approach for a fractional order ODE as follows [40]:

Let an ODE that is fractional.

$${}^{ABC}{℘}_{t}^{\gamma }\varsigma (t)=\psi (t,\varsigma (t))\;\mathrm{With}\;\varsigma (0)=\varsigma_{0}$$ (3)

Eq (3) has the following numerical structure [40]:

$$\begin{array}{l} \varsigma_{r+1}=\varsigma_{0}+\frac{1-\gamma }{N(\gamma )}\psi (t_{r},\varsigma (t_{r}))\\ +\frac{\gamma }{N(\gamma )}\sum_{w=0}^{r}\left[\begin{array}{l} \frac{h^{\gamma }\psi (t_{w},\varsigma (t_{w}))}{\Gamma (\gamma +2)}\{{\left(r+1-w\right)}^{\gamma }(r+2-w+\gamma )-{\left(r-w\right)}^{\gamma }(r+2-w+2\gamma )\}\\ -\frac{h^{\gamma }\psi (t_{w},\varsigma (t_{w}))}{\Gamma (\gamma +2)}\{{\left(r+1-w\right)}^{\gamma +1}-{\left(r-w\right)}^{\gamma }(r+1-w+2\gamma )\} \end{array}\right]. \end{array}$$ (4)

**Lemma 2.1**: suppose $\Phi \subset \mathbb{R}\times {\mathbb{C}}^{n}$ is open, $g_{i}\in \mathbb{C}(\Phi ,\mathbb{R}),i=1,2,3,\ldots ,n.$ if $g_{i}|{}_{x_{i}(\tau )=0,X_{\tau }\in {\mathbb{C}}_{+0}^{n}}\geq 0.$ $X_{\tau }={\left(x_{1t},x_{2t},\ldots ..,x_{nt}\right)}^{T},i=1,2,\ldots .,n,$ Then ${\mathbb{C}}_{+0}^{n}\{\phi =(\phi_{1},\phi_{2},\ldots .,\phi_{n}):\phi \in \mathbb{C}([-\upsilon ,0],{\mathbb{R}}_{+0}^{n})\}$ is the following equations have an invariant domain [22].


$$\frac{dx_{i}(t)}{dt}=g_{i}(t,X_{t}),t\geq 0,i=1,2,\ldots ,n,$$ (5)

$$\mathrm{where}\;{\mathbb{R}}_{+0}^{n}\{(x_{1},x_{2},\ldots ..,x_{n}):x_{i}\geq 0,i=1,2,\ldots \ldots ,n\}.$$ (6)

## 3. Mathematical formulation

**Fig 1:**
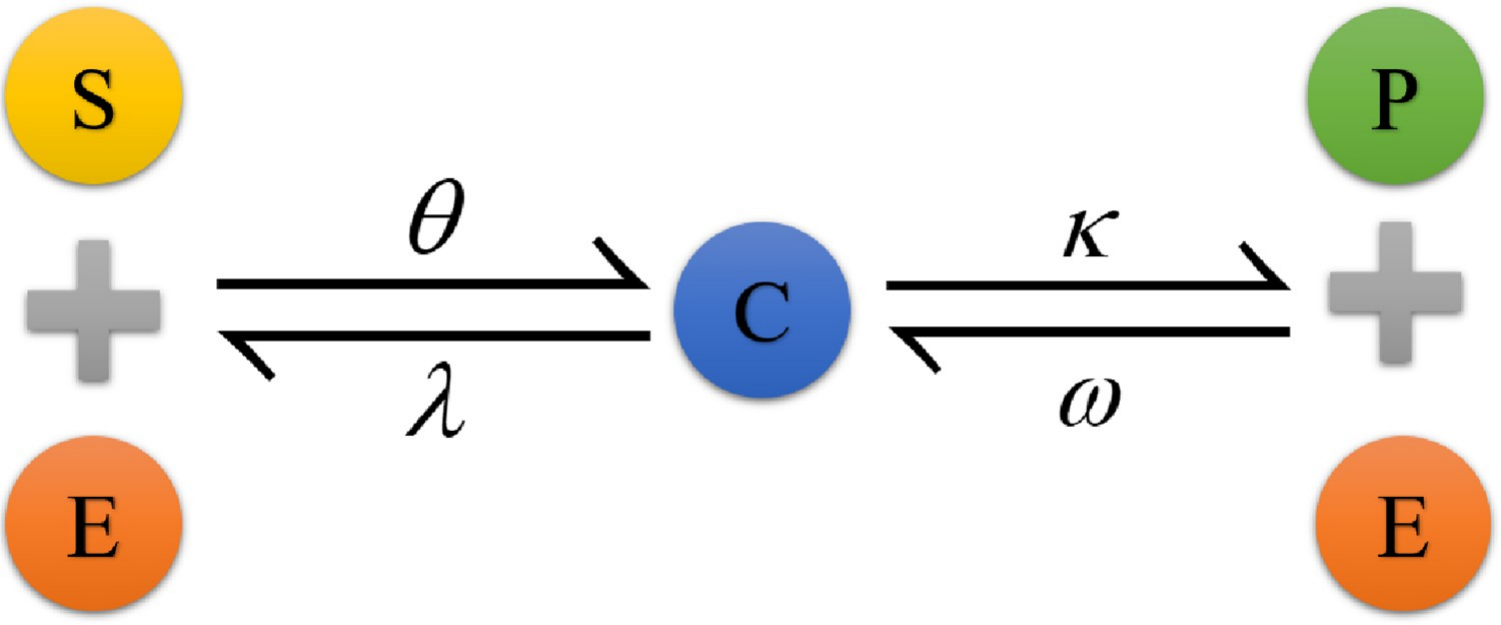
Flow chart for the two-step reversible reaction.

**Fig 2:**
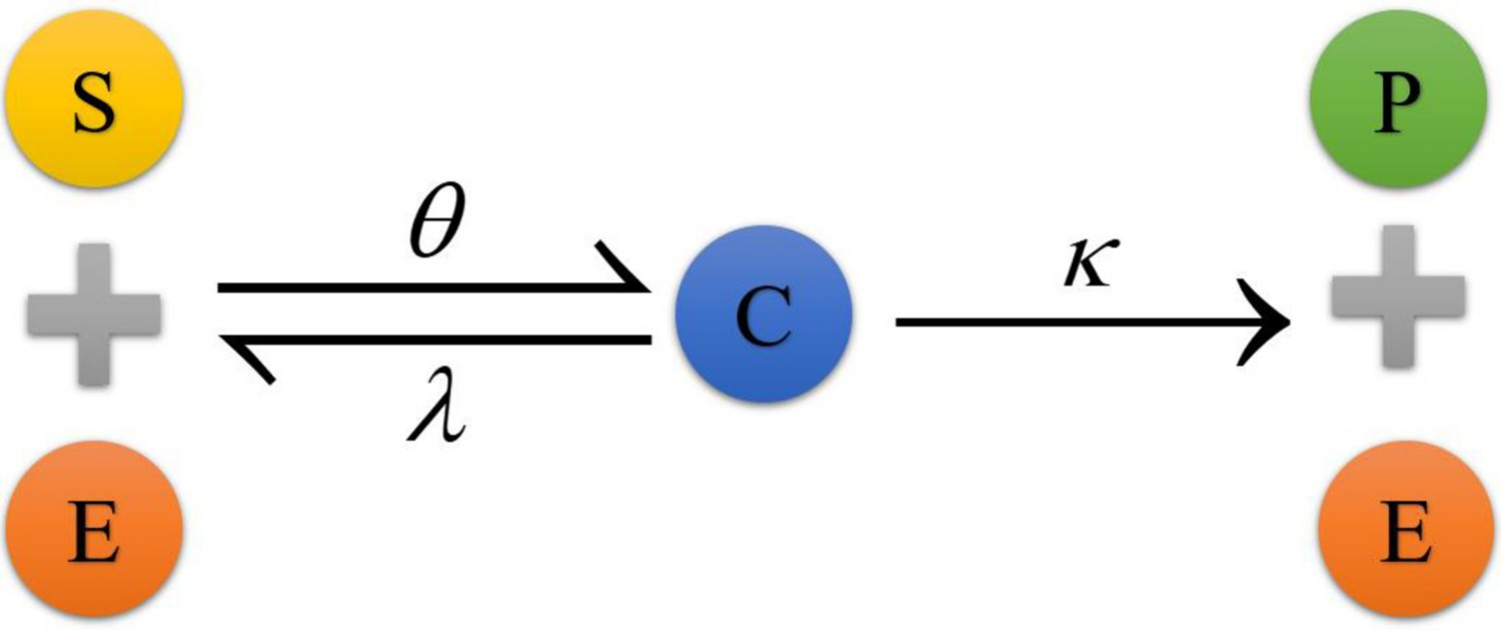
Flow chart of the two-step reaction with one step reversible and one step irreversible.

In this problem, we considered a two-step process where the enzyme *E* reacts with a substrate *S* and then transforms it to the product *P*. To begin, *E* and *S* combine to produce a complex *C* a steady rate that is positive *θ*. This complex *C* then degrades to produce *P*, which releases *E* at a positive rate *κ*, and subsequently to make *S* and *E* again at a positive rate of *λ*. With the backward reaction rate *ω*, some of the parts of *P* and *E* breaking down to produce *C*. The reaction scheme is represented schematically in Fig 1 which is given below [41, 42]:

However, as time passes, the product *P* is constantly eliminated, thus preventing the backward reaction from occurring. As a result, assuming that a response has no reversal rate is usual. As a consequence, the reaction frequently takes the shape seen in Fig 2:

By employing the concept of law of mass action, the abovementioned reactions in mathematical form become [32]:

$$\begin{array}{l} \frac{dS}{dt}=\lambda C-\theta SE,\\ \frac{dE}{dt}=(\lambda +\kappa )C-\theta SE,\\ \frac{dC}{dt}=\theta SE-(\kappa +\lambda )C,\\ \frac{dP}{dt}=\kappa C. \end{array}\}.$$ (7)

With non-negative initial conditions

$$S(0)=s_{0},E(0)=e_{0},C(0)=c_{o},P(0)=p_{0}.$$ (8)

## 4. Fractional Oder chemical kinetics model

The fractionalized of the system (7) with the time-fractional operator is as follows when the ABC time-fractional operator is used, as stated in Eq (1):

$$\begin{array}{l} {}^{ABC}{℘}_{t}^{\gamma }S(t)=\lambda C-\theta SE,\\ {}^{ABC}{℘}_{t}^{\gamma }E(t)=\lambda C+\kappa C-\theta SE,\\ {}^{ABC}{℘}_{t}^{\gamma }C(t)=\theta SE-\kappa C-\lambda C,\\ {}^{ABC}{℘}_{t}^{\gamma }P(t)=\kappa C, \end{array}\},$$ (9)

## 5. Non-negativity of the fractional model

The present part illustrates the non-negativity of system solutions (9). We took notice of this.


$$\begin{array}{l} {}^{ABC}{{℘}_{t}^{\gamma }S(t)|}_{s=0}=\lambda C\geq 0,\\ {}^{ABC}{{℘}_{t}^{\gamma }E(t)|}_{E=0}=\lambda C+\kappa C\geq 0,\\ {}^{ABC}{{℘}_{t}^{\gamma }C(t)|}_{C=0}=\theta SE\geq 0,\\ {}^{ABC}{{℘}_{t}^{\gamma }P(t)|}_{p=0}=\kappa C\geq 0, \end{array}\},$$ (10)

By Lemma 2.1, the present model (9) is non-negative.

## 6. Uniqueness of the AB-time fractional model

The ABC-time fractional model, Eq 3.9, is unique, according to this portion of the study, Eq (3.9) can be expressed by using the ABC integral from Eq (3.2).

$$\begin{array}{l} S(t)-S(0){=}_{0}^{ABC}I_{t}^{\gamma }(\lambda C-\theta SE),\\ E(t)-E(0){=}_{0}^{ABC}I_{t}^{\gamma }(\lambda C+\kappa C-\theta SE),\\ C(t)-C(0){=}_{0}^{ABC}I_{t}^{\gamma }(\theta SE-\kappa C-\lambda C),\\ P(t)-P(0){=}_{0}^{ABC}I_{t}^{\gamma }(\kappa C), \end{array}\},$$ (11)

implying:

$$\begin{array}{l} S(t)-S(0)=\frac{1-\gamma }{N(\gamma )}\varsigma (t,S(t))+\frac{\gamma }{N(\gamma )\Gamma (\gamma )}\int_{0}^{t}\varsigma (\vartheta ,S(\vartheta )){\left(t-\vartheta \right)}^{\gamma -1}d\vartheta ,\\ E(t)-E(0)=\frac{1-\gamma }{N(\gamma )}\varsigma (t,E(t))+\frac{\gamma }{N(\gamma )\Gamma (\gamma )}\int_{0}^{t}\varsigma (\vartheta ,E(\vartheta )){\left(t-\vartheta \right)}^{\gamma -1}d\vartheta ,\\ C(t)-C(0)=\frac{1-\gamma }{N(\gamma )}\varsigma (t,C(t))+\frac{\gamma }{N(\gamma )\Gamma (\gamma )}\int_{0}^{t}\varsigma (\vartheta ,C(\vartheta )){\left(t-\vartheta \right)}^{\gamma -1}d\vartheta ,\\ P(t)-P(0)=\frac{1-\gamma }{N(\gamma )}\varsigma (t,P(t))+\frac{\gamma }{N(\gamma )\Gamma (\gamma )}\int_{0}^{t}\varsigma (\vartheta ,P(\vartheta )){\left(t-\vartheta \right)}^{\gamma -1}d\vartheta , \end{array}),$$ (12)

where

$$\begin{array}{l} \varsigma (t,S(t))=\lambda C-\theta SE,\\ \varsigma (t,E(t))=\lambda C+\kappa C-\theta SE,\\ \varsigma (t,C(t))=\theta SE-\kappa C-\lambda C,\\ \varsigma (t,P(t))=\kappa C. \end{array}\}.$$ (13)

If only *S*(*t*), *E*(*t*), *C*(*t*) and *P*(*t*) are the upper bounds, the expressions ς(t,S(t)),ς(t,E(t)),ς(t,C(t)) and *ς*(*t*,*P*(*t*)) are regarded to obey the Lipchitz criterion. If we treat *S*(*t*) and *S*_1_(*t*) as the two functions, we get

$$\|\varsigma (t,S(t))-\varsigma (t,S_{1}(t))\|=\|\lambda C(t)-\theta S_{1}(t)E(t)-\lambda C(t)+\theta S_{1}(t)E(t)\|.$$ (14)

Equivalent to:

$$\|\varsigma (t,S(t))-\varsigma (t,S_{1}(t))\|\leq \chi_{1}\|S(t)-S_{1}(t))\|,$$ (15)

Where *χ*_1_ = ‖*θE*(*t*)‖ is the corresponding Lipchitz Constant

We can also accomplish:

$$\begin{array}{l} \|\varsigma (t,E(t))-\varsigma (t,E_{1}(t))\|\leq \chi_{2}\|E(t)-E_{1}(t))\|,\\ \|\varsigma (t,C(t))-\varsigma (t,C_{1}(t))\|\leq \chi_{3}\|C(t)-C_{1}(t))\|,\\ \|\varsigma (t,P(t))-\varsigma (t,P_{1}(t))\|\leq \chi_{4}\|P(t)-P_{1}(t))\|. \end{array}$$ (16)

As a consequence, the aforementioned function satisfies the Lipchitz criterion with the relevant Lipchitz condition i.e. *χ*_1_,*χ*_2_,*χ*_3_ and *χ*_4_.

Eq (12) might be stated in a recursive manner as:

$$\begin{array}{l} S_{m}(t)=S(0)+\frac{1-\gamma }{N(\gamma )}\varsigma (t,S_{m-1}(t))+\frac{\gamma }{N(\gamma )\Gamma (\gamma )}\int_{0}^{t}\varsigma (\vartheta ,S_{m-1}(t)){\left(t-\vartheta \right)}^{\gamma -1}d\vartheta ,\\ E_{m}(t)=E(0)+\frac{1-\gamma }{N(\gamma )}\varsigma (t,E_{m-1}(t))+\frac{\gamma }{N(\gamma )\Gamma (\gamma )}\int_{0}^{t}\varsigma (\vartheta ,E_{m-1}(t)){\left(t-\vartheta \right)}^{\gamma -1}d\vartheta ,\\ C_{m}(t)=C(0)+\frac{1-\gamma }{N(\gamma )}\varsigma (t,C_{m-1}(t))+\frac{\gamma }{N(\gamma )\Gamma (\gamma )}\int_{0}^{t}\varsigma (\vartheta ,C_{m-1}(t)){\left(t-\vartheta \right)}^{\gamma -1}d\vartheta ,\\ P_{m}(t)=P(0)+\frac{1-\gamma }{N(\gamma )}\varsigma (t,P_{m-1}(t))+\frac{\gamma }{N(\gamma )\Gamma (\gamma )}\int_{0}^{t}\varsigma (\vartheta ,P_{m-1}(t)){\left(t-\vartheta \right)}^{\gamma -1}d\vartheta . \end{array}\}.$$ (17)

We can achieve the following result by eliminating the difference between two consecutive terms:

$$\left\{ \begin{array}{l} {ϒ}_{s,m}(t)=S_{m}(t)-S_{m-1}(t)=\frac{1}{N(\gamma )}\left(\begin{array}{l} (1-\gamma )\{\varsigma (t,S_{m-1}(t))-\varsigma (t,S_{m-2}(t))\}\\ +\frac{\gamma }{\Gamma (\gamma )}\int_{0}^{t}\left\{\varsigma (\vartheta ,S_{m-1}(\vartheta ))-\varsigma (\vartheta ,S_{m-2}(\vartheta ))\right\}{\left(t-\vartheta \right)}^{\gamma -1}d\vartheta \end{array}\right),\\ {ϒ}_{E,m}(t)=E_{m}(t)-E_{m-1}(t)=\frac{1}{N(\gamma )}\left(\begin{array}{l} \left(1-\gamma )\{\varsigma (t,E_{m-1}(t))-\varsigma (t,E_{m-2}(t))\right\}\\ +\frac{\gamma }{\Gamma (\gamma )}\int_{0}^{t}\left\{\varsigma (\vartheta ,E_{m-1}(\vartheta ))-\varsigma (\vartheta ,E_{m-2}(\vartheta ))\right\}{\left(t-\vartheta \right)}^{\gamma -1}d\vartheta \end{array}\right),\\ {ϒ}_{C,m}(t)=C_{m}(t)-C_{m-1}(t)=\frac{1}{N(\gamma )}\left(\begin{array}{l} \left(1-\gamma )\{\varsigma (t,C_{m-1}(t))-\varsigma (t,C_{m-2}(t))\right\}\\ +\frac{\gamma }{\Gamma (\gamma )}\int_{0}^{t}\left\{\varsigma (\vartheta ,C_{m-1}(\vartheta ))-\varsigma (\vartheta ,C_{m-2}(\vartheta ))\right\}{\left(t-\vartheta \right)}^{\gamma -1}d\vartheta \end{array}\right),\\ {ϒ}_{P,m}(t)=P_{m}(t)-P_{m-1}(t)=\frac{1}{N(\gamma )}\left(\begin{array}{l} \left(1-\gamma )\{\varsigma (t,P_{m-1}(t))-\varsigma (t,P_{m-2}(t))\right\}\\ +\frac{\gamma }{\Gamma (\gamma )}\int_{0}^{t}\left\{\varsigma (\vartheta ,P_{m-1}(\vartheta ))-\varsigma (\vartheta ,P_{m-2}(\vartheta ))\right\}{\left(t-\vartheta \right)}^{\gamma -1}d\vartheta \end{array}\right), \end{array}\right.$$ (18)

It’s imperative to notify that

$$S_{m}(t)=\sum_{k=0}^{m}{ϒ}_{s,k}(t),E_{m}(t)=\sum_{k=0}^{m}{ϒ}_{E,k}(t),C_{m}(t)=\sum_{k=0}^{m}{ϒ}_{C,k}(t),P_{m}(t)=\sum_{k=0}^{m}{ϒ}_{P,k}(t),$$

Moreover, using Eqs (15) and (16) and taking.


$$\begin{array}{l} {ϒ}_{S,m}(t)=S_{m-1}(t)-S_{m-2}(t),{ϒ}_{E,m}(t)=E_{m-1}(t)-E_{m-2}(t),{ϒ}_{C,m}(t)=C_{m-1}(t)-C_{m-2}(t),\\ {ϒ}_{P,m}(t)=P_{m-1}(t)-P_{m-2}(t), \end{array}$$

The following results have been obtained.


$$\begin{array}{l} \|{ϒ}_{S,m}(t)\|\leq \frac{1-\gamma }{N(\gamma )}\chi_{1}\|{ϒ}_{S,m-1}(t)\|+\frac{\gamma }{N(\gamma )\Gamma (\gamma )}\int_{0}^{t}\chi_{1}\|{ϒ}_{S,m-1}(\vartheta )\|{\left(t-\vartheta \right)}^{\beta -1}d\vartheta ,\\ \|{ϒ}_{E,m}(t)\|\leq \frac{1-\gamma }{N(\gamma )}\chi_{1}\|{ϒ}_{E,m-1}(t)\|+\frac{\gamma }{N(\gamma )\Gamma (\gamma )}\int_{0}^{t}\chi_{1}\|{ϒ}_{E,m-1}(\vartheta )\|{\left(t-\vartheta \right)}^{\beta -1}d\vartheta ,\\ \|{ϒ}_{C,m}(t)\|\leq \frac{1-\gamma }{N(\gamma )}\chi_{1}\|{ϒ}_{C,m-1}(t)\|+\frac{\gamma }{N(\gamma )\Gamma (\gamma )}\int_{0}^{t}\chi_{1}\|{ϒ}_{C,m-1}(\vartheta )\|{\left(t-\vartheta \right)}^{\beta -1}d\vartheta ,\\ \|{ϒ}_{P,m}(t)\|\leq \frac{1-\gamma }{N(\gamma )}\chi_{1}\|{ϒ}_{P,m-1}(t)\|+\frac{\gamma }{N(\gamma )\Gamma (\gamma )}\int_{0}^{t}\chi_{1}\|{ϒ}_{P,m-1}(\vartheta )\|{\left(t-\vartheta \right)}^{\gamma -1}d\vartheta , \end{array}\},$$ (19)

**Theorem:** *The current fractional model (9) offers a distinct solution for t*∈[0,*T*] *if the following criterion is met*:

$$\frac{1-\gamma }{N(\gamma )}\chi_{k}+\frac{\gamma }{N(\gamma )\Gamma (\gamma )}\chi_{k}b^{\gamma }<1,k=1,2,3,4,5.$$

***Proof*:** As previously stated, *S*(*t*), *E*(*t*), *C*(*t*) and *P*(*t*) are bounded quantities. The Lipchitz condition is also satisfied by *ς*(*S*(*t*), *t*), *ς*(*E*(*t*), *t*), *ς*(*C*(*t*), *t*) and *ς*(*P*(*t*), *t*), as demonstrated in Eqs (15) and (16). As a result of applying the recursive application, Eq (19) has the following form.


$$\begin{array}{l} \|{ϒ}_{S,m}(t)\|\leq \|S_{0}(t)\|{\left(\frac{\left(1-\gamma \right)}{N(\gamma )}\chi_{1}+\frac{b^{\gamma }}{N(\gamma )\Gamma (\gamma )}\chi_{1}\right)}^{m},\\ \|{ϒ}_{E,m}(t)\|\leq \|E_{0}(t)\|{\left(\frac{\left(1-\gamma \right)}{N(\gamma )}\chi_{2}+\frac{b^{\gamma }}{N(\gamma )\Gamma (\gamma )}\chi_{2}\right)}^{m},\\ \|{ϒ}_{C,m}(t)\|\leq \|C_{}{}_{0}(t)\|{\left(\frac{\left(1-\gamma \right)}{N(\gamma )}\chi_{3}+\frac{b^{\gamma }}{N(\gamma )\Gamma (\gamma )}\chi_{3}\right)}^{m},\\ \|{ϒ}_{P,m}(t)\|\leq \|P_{0}(t)\|{\left(\frac{\left(1-\gamma \right)}{N(\gamma )}\chi_{4}+\frac{b^{\gamma }}{N(\gamma )\Gamma (\gamma )}\chi_{4}\right)}^{m}, \end{array}\},$$ (20)

Therefore, the above-mentioned sequences exist and satisfy $\|{ϒ}_{S,m}(t)\|\to 0$, $\|{ϒ}_{E,m}(t)\|\to 0$, $\|{ϒ}_{C,m}(t)\|\to 0$ and $\|{ϒ}_{P,m}(t)\|\to 0$ as *m*→∞. Further, by applying the triangular inequality for any *j*, we get:

$$\begin{array}{l} \|S_{m+j}(t)-S_{m}(t)\|\leq \sum_{k=m+1}^{m+j}{ℑ}_{1}^{k}=\frac{{ℑ}_{1}^{m+1}-{ℑ}_{1}^{m+j+1}}{1-{ℑ}_{1}},\\ \|E_{m+j}(t)-E_{m}(t)\|\leq \sum_{k=m+1}^{m+j}{ℑ}_{2}^{k}=\frac{{ℑ}_{2}^{m+1}-{ℑ}_{2}^{m+j+1}}{1-{ℑ}_{2}},\\ \|C_{m+j}(t)-C_{m}(t)\|\leq \sum_{k=m+1}^{m+j}{ℑ}_{3}^{k}=\frac{{ℑ}_{3}^{m+1}-{ℑ}_{3}^{m+j+1}}{1-{ℑ}_{3}},\\ \|P_{m+j}(t)-P_{m}(t)\|\leq \sum_{k=m+1}^{m+j}{ℑ}_{4}^{k}=\frac{{ℑ}_{4}^{m+1}-{ℑ}_{4}^{m+j+1}}{1-{ℑ}_{4}}. \end{array}\}.$$ (21)

Where $\frac{\left(1-\gamma \right)}{N(\gamma )}\chi_{k}+\frac{1}{N(\gamma )\Gamma (\gamma )}\chi_{k}b^{\gamma }<1$ by assertion and ${ℑ}_{1},{ℑ}_{2},{ℑ}_{3},{ℑ}_{4}$ Additionally, these terms are enclosed in brackets in equation (20). Thus *S*_*m*_, *E*_*m*_, *C*_*m*_ and *P*_*m*_ denotes the Cauchy sequences in *B*(*ϖ*). They’re all uniformly convergent as a result [18]. When *m*→∞ is applied to Eq (17), The model (9) unique solution clearly restricts these sequences. Unique system solution (9).

## 7. Reaction speed

A reaction speed for the present chemical reaction can be expressed as [42]:

$$V=\frac{\theta \kappa e_{0}S}{\lambda +\theta S}.$$ (22)

The expression above can be written as follows:

$$V=\frac{\theta V_{\max}S}{\lambda +\theta S},$$ (23)

The maximum reaction velocity is *V*_max_ = *κe*_0_ when the enzyme is complexed in combination with the substrate.

## 8. Numerical scheme for the fractional model

The numerical approach is represented as follows using model (9). We might express it as follows in model (9) based on the methods provided in definition 2.3:

$$\begin{array}{l} {}_{0}^{ABC}{℘}_{t}^{\gamma }S(t)=\varsigma (t,S(t)),\\ {}_{0}^{ABC}{℘}_{t}^{\gamma }E(t)=\varsigma (t,E(t)),\\ {}_{0}^{ABC}{℘}_{t}^{\gamma }C(t)=\varsigma (t,C(t)),\\ {}_{0}^{ABC}{℘}_{t}^{\gamma }P(t)=\varsigma (t,P(t)), \end{array}\}.$$ (24)

where

$$\begin{array}{l} \varsigma (t,S(t))=\lambda C-\theta SE,\\ \varsigma (t,E(t))=\lambda C+\kappa C-\theta SE,\\ \varsigma (t,C(t))=\theta SE-\kappa C-\lambda C,\\ \varsigma (t,P(t))=\kappa C. \end{array}\}.$$ (25)

Which implies

$$\begin{array}{l} S(t_{r+1})=S(t_{0})+\frac{1-\gamma }{N(\gamma )}\varsigma (t_{r},S(t_{r}))\\ +\frac{\gamma }{N(\gamma )}\sum_{w=0}^{r}\left[\begin{array}{l} \frac{h^{\gamma }\varsigma (t_{w},S(t_{w}))}{\Gamma (\gamma +2)}\{{\left(r+1-w\right)}^{\gamma }(r+2-w+\gamma )-{\left(r-w\right)}^{\gamma }(r+2-w+2\gamma )\}\\ -\frac{h^{\gamma }\varsigma (t_{w-1},S(t_{w-1}))}{\Gamma (\gamma +2)}\{{\left(r+1-w\right)}^{\gamma +1}-{\left(r-w\right)}^{\gamma }(r+1-w+2\gamma )\} \end{array}\right], \end{array}$$ (26)

$$\begin{array}{l} E(t_{r+1})=E(t_{0})+\frac{1-\gamma }{N(\gamma )}\varsigma (t_{r},E(t_{r}))\\ +\frac{\gamma }{N(\gamma )}\sum_{w=0}^{r}\left[\begin{array}{l} \frac{h^{\gamma }\varsigma (t_{w},E(t_{w}))}{\Gamma (\gamma +2)}\{{\left(r+1-w\right)}^{\gamma }(r+2-w+\gamma )-{\left(r-w\right)}^{\gamma }(r+2-w+2\gamma )\}\\ -\frac{h^{\gamma }\varsigma (t_{w-1},E(t_{w-1}))}{\Gamma (\gamma +2)}\{{\left(r+1-w\right)}^{\gamma +1}-{\left(r-w\right)}^{\gamma }(r+1-w+2\gamma )\} \end{array}\right], \end{array}$$ (27)

$$\begin{array}{l} C(t_{r+1})=C(t_{0})+\frac{1-\gamma }{N(\gamma )}\varsigma (t_{r},C(t_{r}))\\ +\frac{\gamma }{N(\gamma )}\sum_{w=0}^{r}\left[\begin{array}{l} \frac{h^{\gamma }\varsigma (t_{w},C(t_{w}))}{\Gamma (\gamma +2)}\{{\left(r+1-w\right)}^{\gamma }(r+2-w+\gamma )-{\left(r-w\right)}^{\gamma }(r+2-w+2\gamma )\}\\ -\frac{h^{\gamma }\varsigma (t_{w-1},C(t_{w-1}))}{\Gamma (\gamma +2)}\{{\left(r+1-w\right)}^{\gamma +1}-{\left(r-w\right)}^{\gamma }(r+1-w+2\gamma )\} \end{array}\right], \end{array}$$ (28)

$$\begin{array}{l} P(t_{r+1})=P(t_{0})+\frac{1-\gamma }{N(\gamma )}\varsigma (t_{r},P(t_{r}))\\ +\frac{\gamma }{N(\gamma )}\sum_{w=0}^{r}\left[\begin{array}{l} \frac{h^{\gamma }\varsigma (t_{w},P(t_{w}))}{\Gamma (\gamma +2)}\{{\left(r+1-w\right)}^{\gamma }(r+2-w+\gamma )-{\left(r-w\right)}^{\gamma }(r+2-w+2\gamma )\}\\ -\frac{h^{\gamma }\varsigma (t_{w-1},P(t_{w-1}))}{\Gamma (\gamma +2)}\{{\left(r+1-w\right)}^{\gamma +1}-{\left(r-w\right)}^{\gamma }(r+1-w+2\gamma )\} \end{array}\right]. \end{array}$$ (29)

## 9. Graphical results and discussion

**Fig 3:**
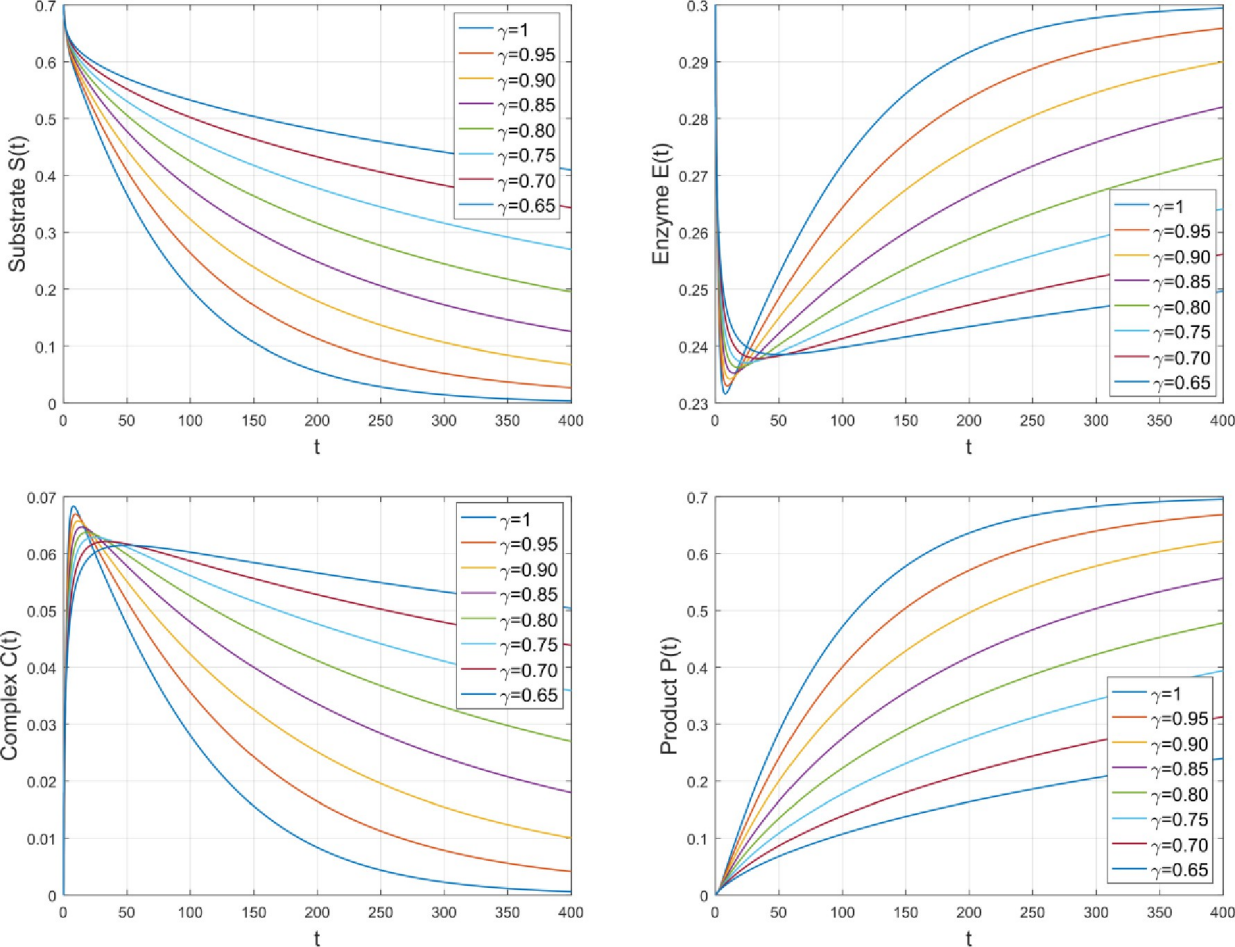
The impact of *γ* on the amount of various species.

The graphical findings of the current research are presented in this portion of the paper. The results of the enzymatic kinetics analysis are presented in this paper. The time-fractional derivative is investigated using the Atangana-Baleanu time-fractional operator of the fractional parameter *γ*. The results are obtained by the use of a numerical technique and the MATLAB programme. For initial conditions, we have considered the arbitrary values i.e., *S*(0) = 0.7, *E*(0) = 0.3 and *C*(0) = *P*(0) = 0. The following is a discussion of the impact of several physical parameters on chemical dynamics:

Fig 3 shows how fractional parameter *γ* affect the concentration effect field of various species. The advantage of considering the fractional model is that it provides us with many solutions as compared to classical models, which have only one solution for integer order. We can get the best match between theoretical and real data by adjusting the fractional parameter appropriately. The considered problem based on enzymatic kinetics can be better explained through the Atangana-Baleanu fractional differential operator. In order to see the significant difference obviously, the simulation is displayed for both fractional order (0<*γ*<1) and integer-order (*γ* = 1). It is important to mention here that by taking *γ*→1, As a result of the reduction to the classical order, we examined the fractional model under consideration.

**Fig 4:**
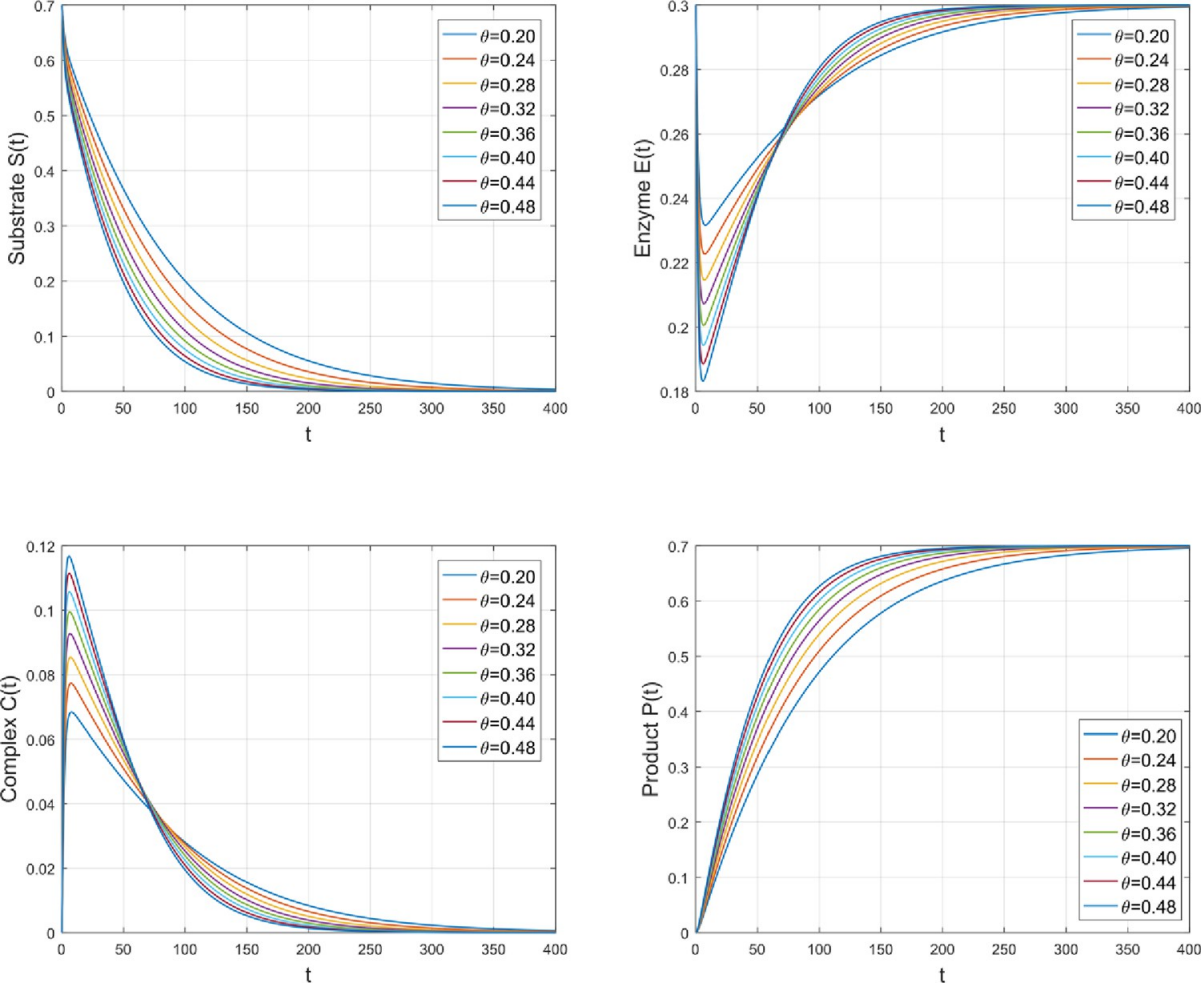
The impact of *θ* on the amount of various species.

Reaction rate *θ* affects the quantity of various species, as seen in Fig 4. The pace at which substrate *S* and enzyme *E* interact to form complex *C* is called the forward reaction rate *θ*. The reaction of *S* with *E* grows as the rate of *θ* increases. The strong interaction between *S* and *E* causes a sufficient amount of *S* and *E* to be converted into a complex *C*. Following that, *C* is distorted into product *P* and enzyme. Therefore, by rising *θ*, the concentration profile of *S* and *E* decreases while *C* and *P* increases for higher values of *θ*.

**Fig 5:**
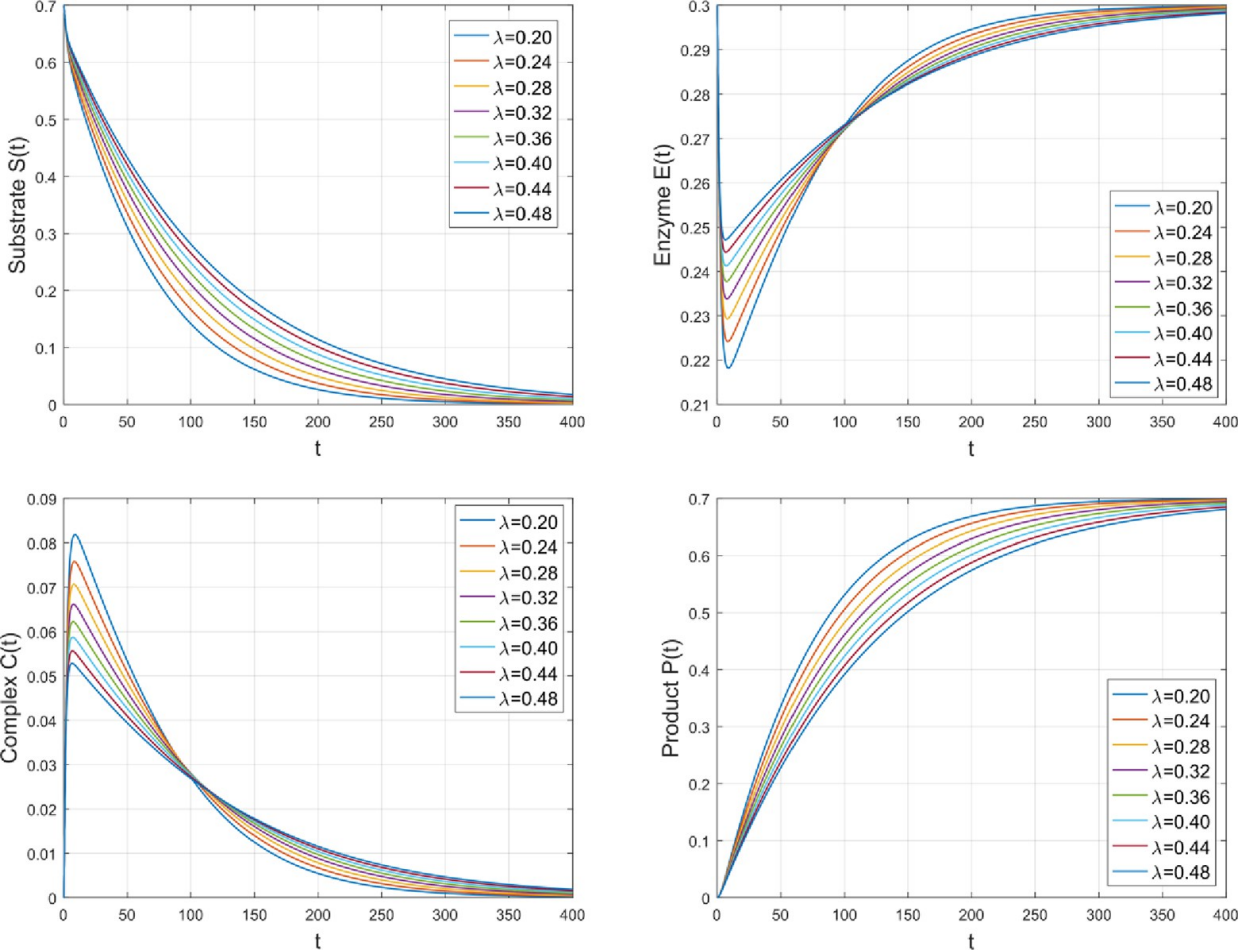
The impact of *λ* on the amount of various species.

The effect of *λ* on the amount of several species is seen in Fig 5. As *λ* is the rate of reversal of deformation of *C* into *S* and *E*. By rising *λ*, the backward deformation rate of *C* increases which increases the concentration distributions of *S* and *E* while *C* and *P* retards for greater values of *λ*.

**Fig 6:**
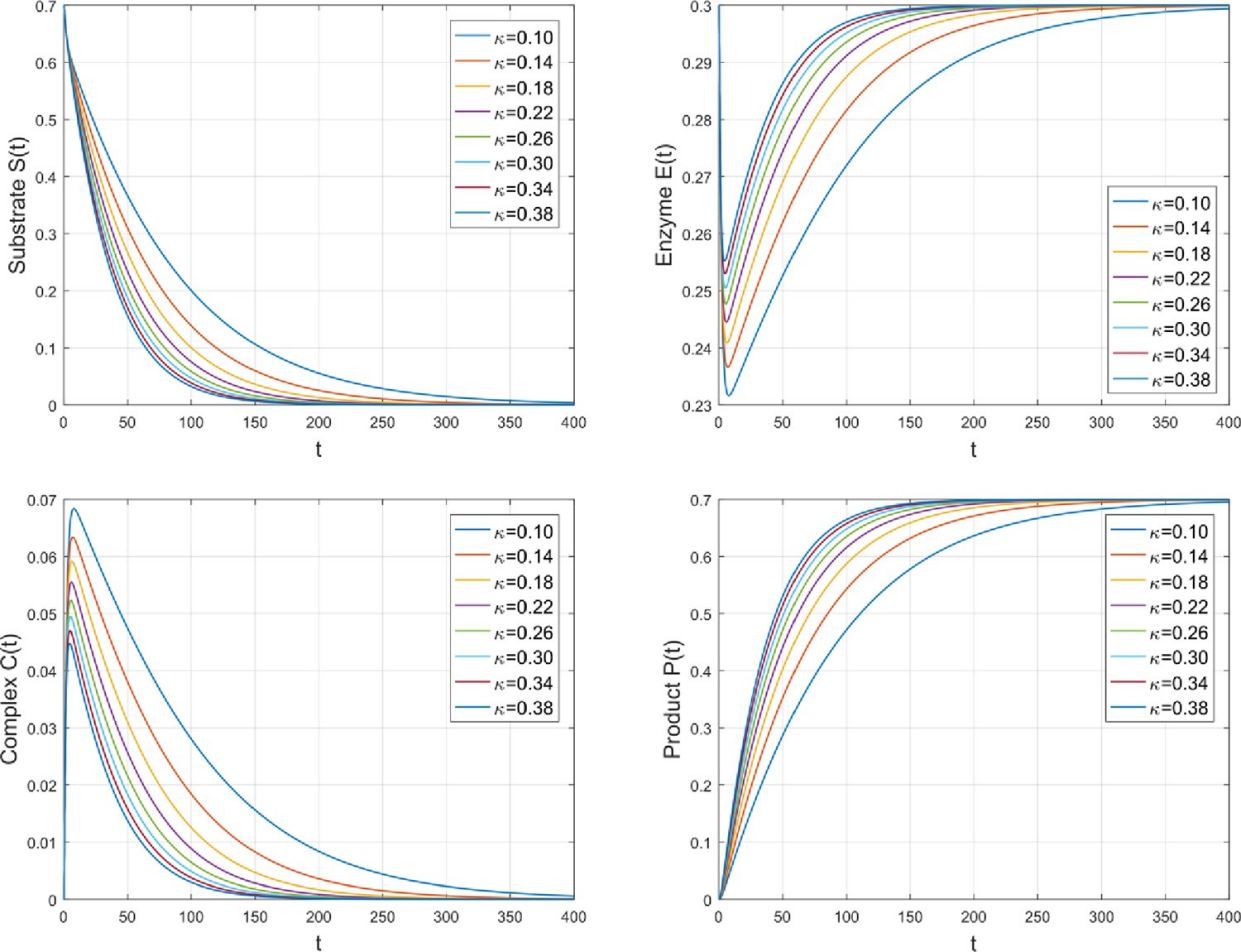
The impact of *κ* on the amount of various species.

The effect of *κ* on the concentration distributions of several species is seen in Fig 6. *κ* is the forward deformation rate of *C* into *P* and *E*. By rising *κ*, it means that forward deformation of *C* increases which increases the concentration profiles of *P* and *E* while in the same ratio, *C* and *S* decrease because there will be no enough amount of *C* to deform back into *S*.

**Fig 7:**
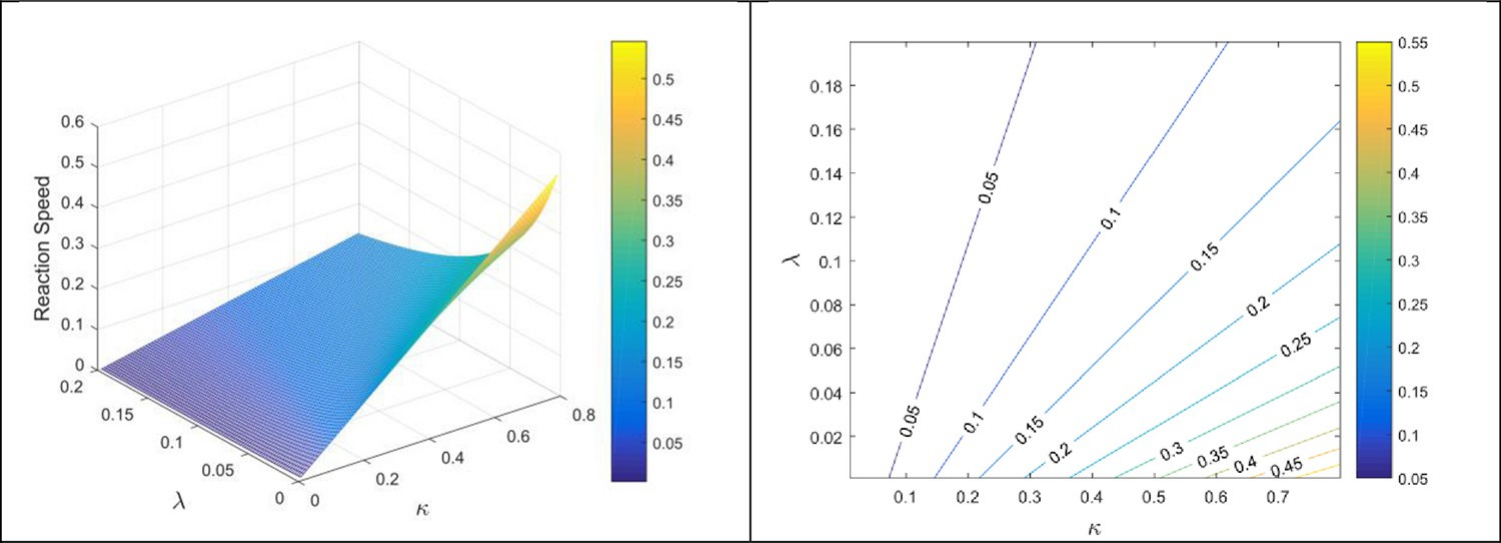
Reaction velocity against *κ* and *λ*.

**Fig 8:**
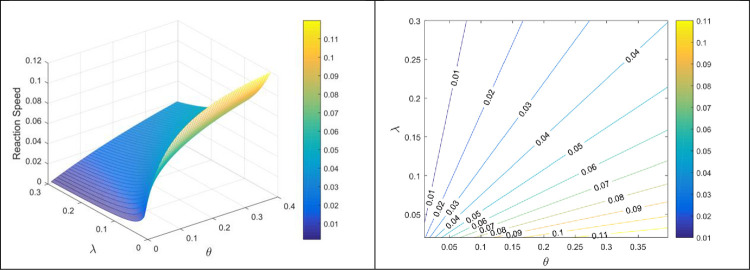
Reaction velocity against *θ* and *λ*.

Figs 7 and 8 displays the reaction speed against different reaction rates. The reaction speed is displayed through 3D portraits and 2D contour graphs. From these figures, it can be noticed that reaction speed increases by increasing forward rates i.e., *κ* and *θ* while the speed reduces with the increasing values of backward reaction rate *λ*. As, these all are physical parameters and if we increase the forward rate by means of some heat or other source, we can get more product in less time. The continuous elimination of product will also stop the backward reaction and the complete reaction will terminate easily with less time consumption.

## 8. Concluding remarks

The dynamics of the enzymatic reaction of chemical kinetics were investigated in the current article. The chemical process was first described using nonlinear classical ODEs, the AB time-fractional operator was utilized to extend the classical model into a fractional one. The fractional model has been demonstrated to have certain fundamental theoretical analysis such as positivity and uniqueness. The expression for reaction speed is also given for the present reaction kinetics. Using the software program MATLAB, numerical approaches have also been used to solve the nonlinear coupled ODEs. The numerical approaches used to solve the given fractional model are briefly discussed. The graphs representing some of the study most fruitful findings are examined. According to the current investigation, forward reaction rates improve reaction speed, however reverse deformation of complex enzyme slows down the entire process as shown in the figures. By increasing forward reaction through different means such as heating or adding a sufficient amount of catalyst can increase the reaction speed through which we can produce more product in less time.

In future, the current work can be extended into different directions like the consideration of these chemical reactions in different plants and animal cells or the reations on the industrial scale. The role of ATP can also be introduce to such kind of problems. Rather than that, the reactions in the big reactors can also be modeled through the considered approach. The existing models can be resolved by implementing the fractal-fractional differential operators of Mittag-Leffler, exponential or power-law kernels in order to include memory and fractal behavior at once because no one implemented the fractal-fractional operators on such kind of biochemical reaction problems.
